# Assessing the Impact of Frailty on Infection Risk in Older Adults: Prospective Observational Cohort Study

**DOI:** 10.2196/59762

**Published:** 2024-10-16

**Authors:** Ya Yang, Kechun Che, Jiayan Deng, Xinming Tang, Wenyuan Jing, Xiuping He, Jiacheng Yang, Wenya Zhang, Mingjuan Yin, Congcong Pan, Xiaoling Huang, Zewu Zhang, Jindong Ni

**Affiliations:** 1 School of Public Health, Shunde Women and Children’s Hospital, Precision Key Laboratory of Public Health, Guangdong Medical University, No.1 Xincheng Road, Dongguan, 523808, China, 86 15817668208; 2 Office of Public Health, Songshan Lake Community Health Service Centre, DongGuan, China; 3 Institute for Infectious Disease Prevention and Control, DongGuan Centre for Disease Control and Prevention, DongGuan, China

**Keywords:** community elderly, frailty, infectious diseases, infectious disease, older, China, questionnaire, survey, cohort, COVID-19, infection, chi-square, longitudinal analysis, age-related chronic disease, chronic disease, chronic diseases

## Abstract

**Background:**

Infectious diseases are among the leading causes of death and disability and are recognized as a major cause of health loss globally. At the same time, frailty as a geriatric syndrome is a rapidly growing major public health problem. However, few studies have investigated the incidence and risk of infectious diseases in frail older people. Thus, research on frailty and infectious diseases is urgently needed.

**Objective:**

The purpose of this study was to evaluate the association between frailty and infectious diseases among older adults aged 65 years and older.

**Methods:**

In this prospective observational cohort study, we have analyzed the infectious disease prevalence outcomes of older adults aged 65 years and older who participated in frailty epidemiological surveys from March 1, 2018, to March 2023 in Dalang Town, Dongguan City, and from March 1, 2020, to March 2023 in Guancheng Street, Dongguan City. This study has an annual on-site follow-up. Incidence data for infectious diseases were collected through the Chinese Disease Control and Prevention Information System—Infectious Disease Monitoring and Public Health Emergency Monitoring System. A project-developed frailty assessment scale was used to assess the frailty status of study participants. We compared the incidence rate ratios (IRR) of each disease across frailty status, age, and gender to determine the associations among frailty, gender, age, and infectious diseases. Cox proportional hazards regression was conducted to identify the effect of frailty on the risk of demographic factors and frailty on the risk of infectious diseases, with estimations of the hazard ratio and 95% CI.

**Results:**

A total of 235 cases of 12 infectious diseases were reported during the study period, with an incidence of 906.21/100,000 person-years in the frailty group. In the same age group, the risk of infection was higher in men than women. Frail older adults had a hazard ratio for infectious diseases of 1.50 (95% CI 1.14‐1.97) compared with healthy older adults. We obtained the same result after sensitivity analyses. For respiratory tract–transmitted diseases (IRR 1.97, 95% CI 1.44‐2.71) and gastrointestinal tract–transmitted diseases (IRR 3.67, 95% CI 1.39‐10.74), frail older adults are at risk. Whereas no significant association was found for blood-borne, sexually transmitted, and contact-transmitted diseases (IRR 0.76, 95% CI 0.37‐1.45).

**Conclusions:**

Our study provides additional evidence that frailty components are significantly associated with infectious diseases. Health care professionals must pay more attention to frailty in infectious disease prevention and control.

## Introduction

Infectious diseases manifest through the invasion of pathogenic microorganisms that disrupt the normal physiological functions of the host, leading to adverse effects and dysfunction [1]. Evidence suggests that airborne and droplet-borne infectious diseases have inflicted severe socioeconomic costs on regional and global industries [2]. When facing a pathogenic invasion, the immune system is the key to survival. However, the immune system undergoes significant changes with age. Many older people show a decline in immune responses. This has been termed “immunosenescence” and predisposes older people to infections and also to weaker vaccination responses than seen in young and middle-aged adults [3,4]. In addition, disorders of the immune system can lead to vulnerability to infection, cancer, autoimmune illness, and vaccine failure [5]. In comparison to young adults, older adults are more susceptible to infectious diseases due to immunosenescence and poor nutritional status [4,6-10]. The COVID-19 pandemic highlights this issue even further.

As the global population is aging rapidly, frailty in older people is going to be more and more of a concern. Frailty has been defined as a state of increased vulnerability to adverse health outcomes, secondary to multiple deficits in physiological, physical, and mental function [11]. Frail older people are more likely to be infected with fungi, bacteria, and viruses than the general population [12-14]. Frailty has been reported to affect the susceptibility and severity of pneumonia in people older than 65 years of age and is strongly associated with their higher 1-year mortality [15,16]. Studies have shown that frailty is associated with the attenuation of vaccine-induced responses to antibodies and an increase in postvaccination influenza infections among community-dwelling older adults and that vaccine effectiveness declines with increasing frailty and age [17-19]. In addition, there is evidence that those aged 60 years and older are at the highest risk of dying from influenza and that this risk increases with age [20]. A cohort study found an increased risk of developing COVID-19 in the presence of frailty syndromes [21]. However, the association between frailty and infection lacks adequate evidence from existing research.

At present, we are facing the dual threat of emerging and reemerging infectious diseases. As the population ages, a new challenge is surfacing for societies—frailty. Current evidence suggests that frailty increases the risk of developing infections. However, there has been little investigation of this in China. Studying a population-based sample of older adults categorized by their frailty status may help to untangle the relationship between frailty and infection. Thus, we conducted a population-based prospective and observational study focusing on frailty and infection. The results may help to disentangle the association between frailty and infections.

## Methods

### Study Population

This study was based on the Community Elderly Health Survey Follow-up Cohort, which recruited older people aged 65 years and older who received free health checkups at community hospitals, excluding those with Alzheimer disease, and those who were unable to communicate normally due to hearing and vision impairments. This national basic public health service is conducted once a year in community hospitals. Our study participants were from Dalang Town and Guancheng Street in Dongguan City, with the Dalang Town cohort in Dongguan City starting in 2018 and the other cohort starting in 2020. Baseline data were collected in the 2018 and 2020 surveys, and study participants were followed up once a year without interruption, with follow-up ending either at the time of an infectious disease or at the end of the study (March 2023). Physical examinations were conducted by medical staff, and trained investigators administered questionnaires to older adults using a project-developed frailty assessment scale and collected basic information in a one-on-one face-to-face survey, including demographic, lifestyle, and frailty status.

**Figure 1.**
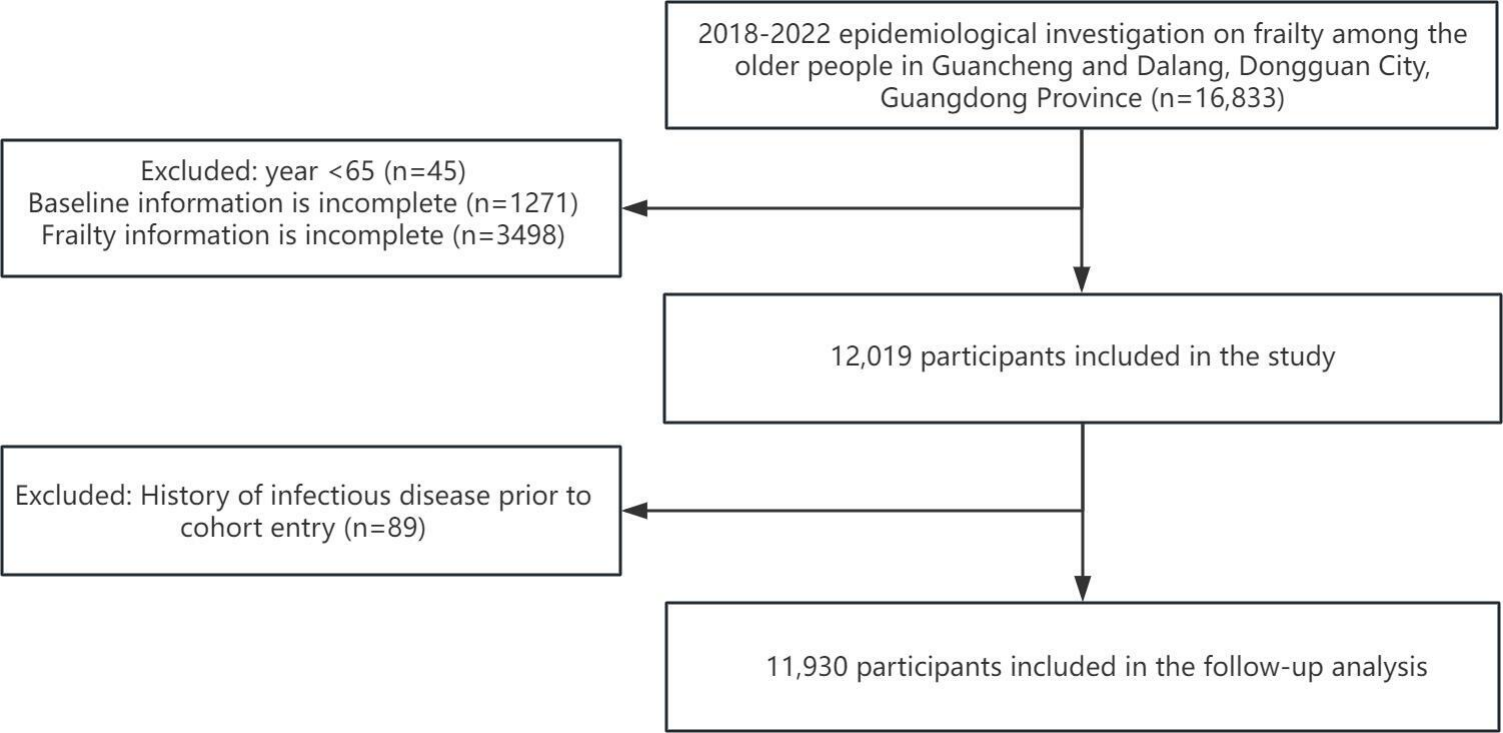
The flowchart of participant inclusion.

A total of 16,833 older adults who met the inclusion criteria were included in the study cohort, none of whom had an infectious disease at baseline. The relationship between frailty and infectious diseases was analyzed for 11,930 participants after excluding 4814 participants with incomplete information and 89 participants who already had an infectious disease before the start of the survey. The flowchart of participant inclusion is shown in Figure 1.

### Ascertainment of Frailty

The diagnostic criteria of the project-developed frailty evaluation scale were used as the basis for diagnosing frailty in this study. The evaluation was graded by calculating the scores of the debility assessment and analysis entries, with a frailty index <0.22 evaluated as healthy and a frailty index ≥0.22 evaluated as frail. The scale is based on the frailty index model and contains 33 entries in 7 dimensions, including general health status, ability to perform activities of daily living, functional activities, symptoms and signs, cognitive functioning, social support, and psychological status. Tests of the older population showed that the internal consistency reliability of the scale with Cronbach α coefficient was 0.87, and the correlation coefficient between the score of the frailty index scale and the score of the frailty phenotype was 0.78 (*P*<.001). As such, its reliability was good [22].

### Ascertainment of Infection

After collecting the baseline information, outcome events were tracked annually through follow-ups on the study’s participants. The outcomes included statutory infectious disease incidence. We used the Chinese Disease Control and Prevention Information System—Infectious Disease Monitoring and Public Health Emergency Monitoring System to query and match people’s statuses, as well as telephone for active follow-up until they experienced infectious disease or until March 30, 2023, whichever came first. The primary outcome of this study was the incidence of infectious diseases. All cases collected were those that were identified by pathogenicity testing and reported to the Chinese Disease Control and Prevention Information System—Infectious Disease Monitoring and Public Health Emergency Monitoring System by medical workers. As of March 2023, it included 2 class A infectious diseases and 27 class B infectious diseases, totaling 40 kinds and excluding mpox, which was newly added in September 2023 [23,24].

### Ascertainment of Covariates

Gender, age, educational level (illiterate, primary school, junior high school, and above), smoking status (never, ever, and yes), drinking alcohol status (everyday, more than once a week, occasional, and never), physical activity level, and participation in community activities were obtained by interviews and physical examinations. Physical activity level was determined by grading the level of physical activity, including low, medium, and high intensity [25]. Participation in community activities refers to participation in activities carried out in the community for the purpose of enriching the spiritual cultural life and social life of the residents, such as badminton, ping-pong, mah-jongg, chess, choir, dance troupes, and so on, including frequent, occasional, and never.

### Sensitivity Analyses

When infected with the hepatitis B virus and hepatitis C virus (HCV), patients do not experience symptoms immediately but rather go through a period of incubation before developing the disease. Hepatitis B carriers have an indeterminate amount of time to develop symptoms and may not even develop the disease. Hepatitis C has an insidious onset, and the initial symptoms are not obvious. So there may be a discrepancy between the time of onset of the disease as detected and the actual time of onset of the disease. In addition, among respiratory tract–transmitted diseases, tuberculosis is a chronic infectious disease. Its early stage often does not have very special symptoms. Patients only have a slight cough or cough sputum, so this symptom is easily ignored by patients and they do not go to see a doctor. It usually has a longer incubation period, sometimes several years or even lifelong. Therefore, the actual onset time of these 3 types of patients is difficult to determine. In order to exclude this part of the impact, we removed the data of these 3 types of patients and reanalyzed the impact of infectious diseases with different transmission routes.

### Ethical Considerations

Ethics approval for this study was sought from the institutional ethics committee of Guangdong Medical University (YJYS2018046). The survey methodology and data collection procedures were approved by the institutional review board of the Guangdong Medical University. Written informed consent was obtained from all participants before recruitment. For secondary analyses of research data, the original informed consent forms included provisions allowing for secondary analysis. All data were anonymously analyzed. Participant received compensation in the form of a gift as an appreciation for their time and contribution to this study.

### Statistical Analysis

First, we conducted a descriptive analysis of the baseline characteristics. The demographics and lifestyles of people at various frailty levels were described. The counting data were presented in frequency and percentage form. Due to the different chronological order of entry into the cohort, each subject was observed for a different period of time. Therefore, person-years of follow-up were calculated on an individual basis. We use the chi-square test to examine the differences between different levels of demographic data (age, gender, and education level), lifestyle (participation in community activities, alcohol consumption, tobacco use, and exercise), and morbidity data (frailty and incidence of infectious diseases). The temporal, regional, and population distributions of each infectious disease were described based on the incidence densities. Incidence rate ratios (IRRs) were used to compare the incidence densities of different populations for the same disease. The 95% CI was used to determine whether the difference was statistically significant.

Next, we compared the risk of enduring infectious disease in 2 levels of frailty. We used the Cox regression model with stepwise adjustment of confounding variables to determine the risk ratio and 95% CI for enduring infectious diseases. Then, we excluded individuals with hepatitis B and C and tuberculosis to conduct sensitivity analyses to test the robustness of the result and to minimize potential reverse causation due to the incubation period. All statistical analyses were performed using SPSS (version 21.0; IBM Corp) and MedCale (version 20.1.4; MedCale Software), with a 2-sided *P* value of <.05 deemed statistically significant.

## Results

### Baseline Information

Baseline information on the study cohort of 11,930 participants is shown in Table 1. During the 35,658 person-years of follow-up, we recorded 235 cases of statutorily reported infectious diseases. The frailty and control groups showed significant differences in age distribution, gender ratio, education level, drinking alcohol status, physical activity level, and participation in community activities, but nonsignificant differences in smoking status.

Statutorily reported infectious diseases were categorized as blood-borne, sexually transmitted and contact-transmitted diseases, natural sources and vector-borne diseases, and gastrointestinal tract–transmitted diseases and respiratory tract–transmitted diseases. A total of 12 statutorily reported infectious diseases were present in the cohort, and none were from natural sources or vector-borne diseases (Table 2).

**Table 1.** Baseline demographics and lifestyle of participants (N=11,930) by frailty status at follow-up.

Factor or group	Total, n (%)	Health, n (%)	Frailty, n (%)	*P* value
**Age (years)**				*<*.001
65	8666 (72.6)	6237 (79.3)	2429 (59.7)	
75	2731 (22.9)	1476 (18.8)	1255 (30.8)	
85	533 (4.5)	148 (1.9)	385 (9.5)	
**Gender**				*<*.001
Men	4851 (40.7)	3547 (45.1)	1304 (32.0)	
Women	7079 (59.3)	4314 (54.9)	2765 (68.0)	
**Education level**				*<*.001
Illiterate	1664 (13.9)	717 (9.1)	947 (23.3)	
Primary school	5492 (46.0)	3650 (46.4)	1842 (45.3)	
Junior high school and above	4774 (40.0)	3494 (44.4)	1280 (31.5)	
**Smoking status**				.34
Never	8445 (70.8)	5534 (70.4)	2911 (71.5)	
Ever	1044 (8.8)	706 (9.0)	338 (8.3)	
Yes	2441 (20.5)	1621 (20.6)	820 (20.2)	
**Drinking alcohol status**				*<*.001
Everyday	349 (2.9)	247 (3.1)	102 (2.5)	
More than once a week	207 (1.7)	141 (1.8)	66 (1.6)	
Occasional	966 (8.1)	727 (9.2)	239 (5.9)	
Never	10,408 (87.2)	6746 (85.8)	3662 (90.0)	
**Physical activity level**				*<*.001
Low intensity	2125 (17.8)	1136 (14.5)	989 (24.3)	
Medium intensity	6790 (56.9)	4524 (57.5)	2266 (55.7)	
High intensity	3015 (25.3)	2201 (28.0)	814 (20.0)	
**Participation in community activities**				*<*.001
Frequent	1898 (15.9)	1345 (17.1)	553 (13.6)	
Occasional	1388 (11.6)	950 (12.1)	438 (10.8)	
Never	8644 (72.5)	5566 (70.8)	3078 (75.6)	

**Table 2.** Disease name and case of the 3 categories of infectious diseases in Dongguan, 2018‐2023.

Classification and name		Values, n
**Respiratory tract–transmitted diseases**		
	Influenza	7
	Mumps	2
	Tuberculosis	15
	COVID-19 infection	142
**Gastrointestinal tract–transmitted**		
	The other infectious diarrhea^a^	21
**Blood-borne, sexually transmitted, and contact-transmitted**		
	HIV	1
	Viral hepatitis (HBV^b^ and HCV)^c^	24
	Syphilis	18
	Condyloma acuminatum	1
	Genital herpes	3
	Chlamydia trachomatis infection of the reproductive tract	1

a Refers to infectious diarrhea other than cholera, dysentery, typhoid, and paratyphoid.

b HBV: hepatitis B virus.

c HCV: hepatitis C virus.

### Frailty of Different Groups

By comparing the incidence of infectious diseases in different groups, we found that the risk of infectious disease increased for people in the frail group by 73% (IRR 1.73, 95% CI 1.33‐2.25; see Table 3). We analyzed the frailty status in 3 groups. The results showed a greater risk of infectious diseases in older adults in the pre-frail and frail groups compared to those in the healthy group (see Table S1 in Multimedia Appendix 1 .

**Table 3.** Person-years, cases, incidence density, and IRR^a^ of different frailty status in a population-based cohort study of older adults in Dongguan, 2018‐2023.

Groups	Total person-years, n	Cases, n	Incidence density (per 100,000 person-years), n	IRR (95% CI)	
Frailty	12,579.8	114	906.21	1.73 (1.33‐2.25)	
Health	23,078.7	121	524.29	1 (reference)	
Total	35,658.5	235	659.03	N/A^b^	

a IRR: incidence rate ratio.

b N/A: not applicable.

### Gender- and Age-Stratified Analyses

The incidence densities of men in all age groups were higher than those of women in the same age group, and the incidence densities of women increased with age (Table 4).

**Table 4.** Person-years, cases, incidence density, and IRR^a^ of infectious disease by gender and age in a population-based cohort study of older adults in Dongguan, 2018‐2023.

Age (years)	Case, n		Person-year, n		Incidence density, n		IRR		95% CI
	Men	Women	Men	Women	Men	Women	Men	Women	
65	62	59	10,091	15,233.30	614.41	387.31	1.59	1.00	1.09‐2.31
75	54	41	3437.90	5163.20	1570.74	794.08	1.98	1.00	1.29‐3.04
85	9	10	634.20	1098.90	1419.08	910	1.56	1.00	0.56‐4.27

a IRR: incidence rate ratio.

### Frailty and Infectious Diseases

Frailty was associated with a higher risk of respiratory tract–transmitted diseases (IRR 1.97, 95% CI 1.44‐2.71) and gastrointestinal tract–transmitted diseases (IRR 3.67, 95% CI 1.39‐10.74; see Table 5).

There were significant differences (*P*<.05) in baseline information between the 2 groups, including gender, age, participation in social activities, educational level, level of physical activity, and drinking alcohol status, as shown in Table 1. As such, it is important to exclude the effects of these confounding factors. The Cox proportional risk model analysis results are presented in Table 6. The risk factors influencing the incidence of infectious diseases were 75 years and older in age, men (hazard ratio [HR] 1.903, 95% CI 1.468‐2.466), never participating in community activities, physical activity other than high intensity, and frailty. Corrected for these factors, frailty status continued to have a significant effect on the incidence of communicable disease in frail older adults (HR 1.50, 95% CI 1.14‐1.97). Again we do the analysis by dividing the frailty status into 3 groups. The results showed that older people in the prefrail group (HR 1.45, 95% CI 1.07‐1.96) and in the frail group (HR 1.65, 95% CI 1.12‐2.44) have a higher risk of infectious diseases than the healthy group (see Table S2 in Multimedia Appendix 1).

The results of the proportional risk model for Cox after adjusting for demographics and behavioral lifestyle after excluding hepatitis B and C and tuberculosis are shown in Table S3 in Multimedia Appendix 1 . The results show that frailty increases the risk of infectious diseases in older adults (HR 1.83, 95% CI 1.36‐2.46). The same conclusion was reached after dividing the frail states into 3 groups (see Table S4 in Multimedia Appendix 1).

**Figure 2.**
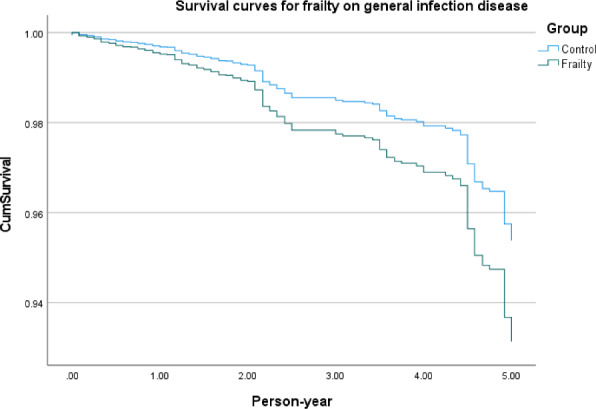
Survival curves for frailty on general infection disease.

**Figure 3.**
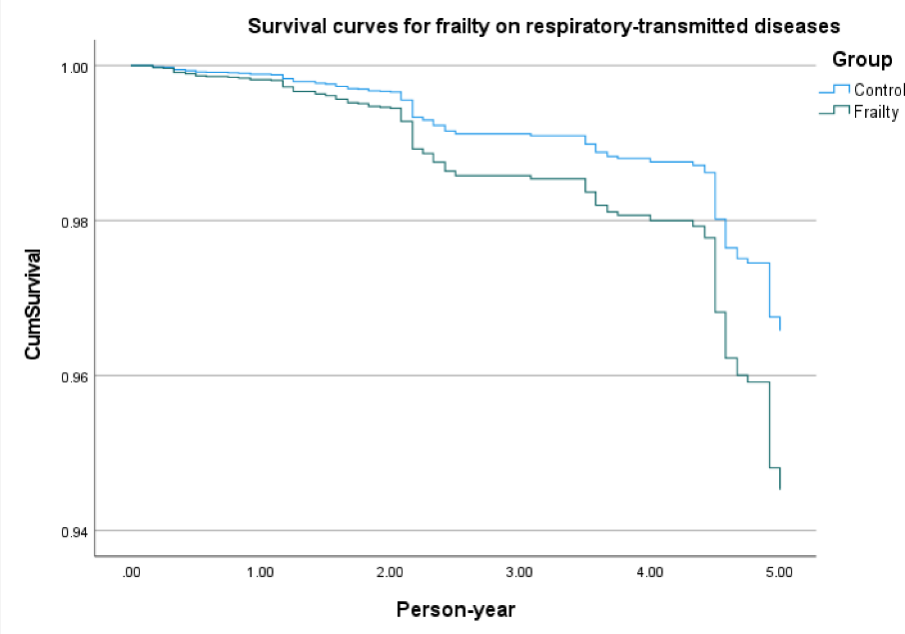
Survival curves for the frailty on respiratory tract–transmitted diseases.

**Figure 4.**
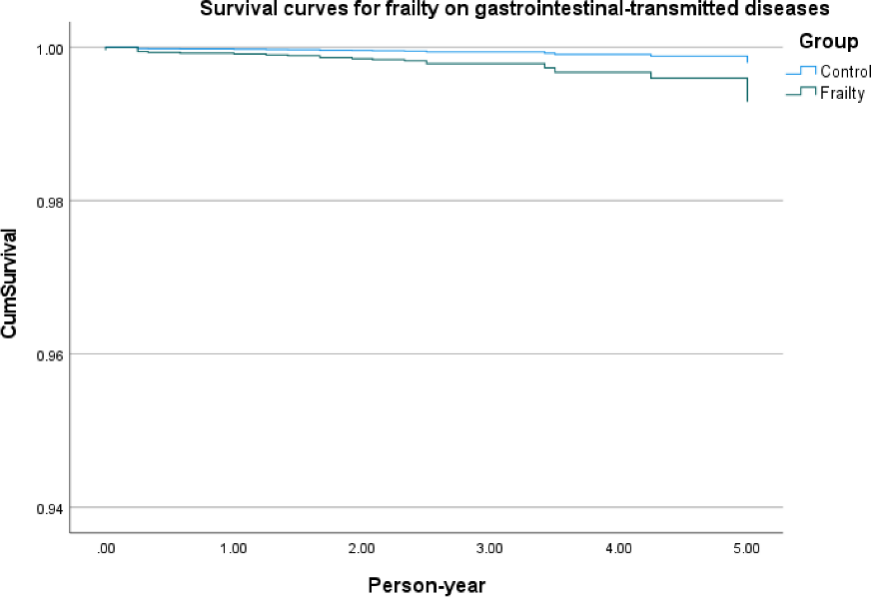
Survival curves for the frailty on gastrointestinal tract–transmitted diseases.

**Figure 5.**
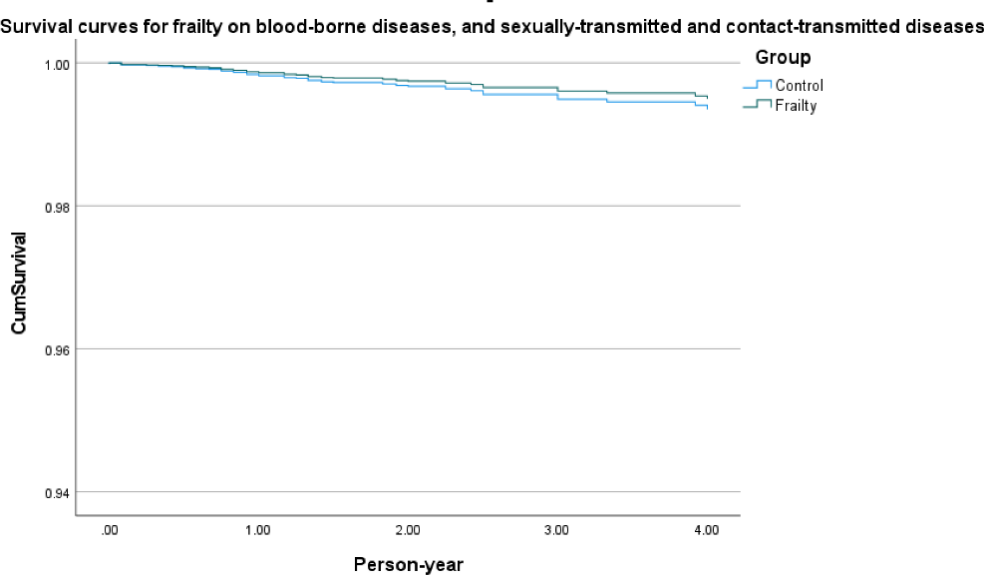
Survival curves for the frailty on blood-borne diseases, and sexually transmitted, and contact-transmitted diseases.

Figure 2 shows survival curves of frailty in infectious diseases in general, frailty in respiratory tract–transmitted diseases (Figure 3), frailty in gastrointestinal tract–transmitted diseases (Figure 4), and frailty in blood-borne diseases, sexually transmitted, and contact-transmitted diseases (Figure 5). It can be seen that frailty has less of an effect on gastrointestinal tract–transmitted, blood-borne, sexually transmitted, and contact-transmitted diseases, while it has some effect on respiratory tract–transmitted diseases.

**Table 5.** Incidence density and IRR^a^ by 3 categories of infectious disease in a population-based cohort study of older adults in Dongguan, 2018‐2023.

Transmission	Incidence density (per 100,000 person-years), n		IRR (95% CI)	
	Frailty	Health		
Respiratory tract–transmitted	683.64	346.64	1.97 (1.44‐2.71)	
Gastrointestinal tract–transmitted	111.29	30.33	3.67 (1.39‐10.74)	
Blood-borne, sexually transmitted, and contact-transmitted	111.29	147.32	0.76 (0.37‐1.45)	

a IRR: incidence rate ratio.

**Table 6.** The outcomes of Cox proportional hazards regression models assessing the association between the frailty and the risk of infectious disease adjusted for age, gender, community participation status, and exercise status (Dongguan; 2018‐2023; N=11,930).

Factor	*P* value	HR^a^ (95% CI)
**Group**		
Health	Reference	
Frailty	.004	1.50 (1.14‐1.97)
**Age (years)**		
65	Reference	
75	<.001	1.97 (1.50‐2.60)
85	.03	1.75 (1.06‐2.90)
**Gender**		
Women	Reference	
Men	<.001	1.92 (1.48‐2.48)
**Participation in community activities**		
Never	Reference	
Frequent	.02	0.65 (0.46‐0.93)
Occasional	.62	1.11 (0.73‐1.70)
**Exercise**		
High intensity	Reference	
Low intensity	.22	1.31 (0.85‐2.00)
Medium intensity	.002	1.74 (1.23‐2.47)

a HR: hazard ratio.

## Discussion

### Principal Findings

This study, which used a prospective cohort study, aimed to explore the association between frailty and the risk of developing infectious diseases in a population of older adults aged 65 years and older. First, we observed a higher risk of developing infectious diseases in frail older adults compared to nonfrail older adults. Second, similar results were observed for respiratory and gastrointestinal transmissible diseases. In addition, we performed sensitivity analyses to further verify the stability of the results. This reliable and robust result can provide intervention strategies for the prevention of infectious diseases in older adults and promote healthy aging in China.

The main finding of this study is that frailty increases the risk of infectious diseases in older adults, independent of age, gender, community activity participation, and exercise. As we age, the aging of the immune system leads to a low-level chronic systemic inflammation state that is basically inactive—called “inflammatory aging” [26]. It has been suggested that so-called “inflammatory aging” may slowly damage 1 or several organs, leading to an increased risk of age-related chronic disease and frailty [27]. Meanwhile, immunesenescence has been associated with increased susceptibility to infectious pathogens and poor vaccine response in older adults [28]. Also, frail older adults have long-standing inflammation compared to the general population [29]. Frailty predisposes older adults to infectious diseases by affecting their regulatory T cells and senescent natural killer cells [30]. The relationship between frailty and increased risk of infectious diseases can be explained in terms of lifestyle behaviors. First, frail older adults are more likely to have physical inactivity and sedentary behaviors [31], and exercise enhances the function of the immune system [32]. In addition, stroke and diabetes have been reported to increase the risk of infectious diseases [33,34], and the risk of infectious diseases is lower in women than in men [35]. Our study found that after adjusting for these variables, frailty still increased the risk of infectious diseases(see Table S5 in Multimedia Appendix 1) . Therefore, in the face of emerging and reemerging infectious diseases, we recommend that frailty identification be included in measures to prevent infectious diseases.

Among respiratory tract–transmitted diseases, we observed an IRR of 1.97 (95% CI 1.44‐2.71) for respiratory tract–transmitted diseases in frail older adults compared with healthy older adults. Previous studies on the association between frailty and respiratory tract–transmitted diseases have focused on individual diseases. Frailty increases the risk of developing COVID-19 and influenza [21,36]. The danger of tuberculosis among older adults has been reported to increase with sarcopenia and physical inactivity, which are major components of the frailty syndrome [37,38]. Although our study centers on the category of respiratory tract–transmitted diseases, our results are consistent with those of prior research. Similarly, frailty has a similar effect on gastrointestinal infectious diseases. However, we only collected other infectious diarrheal diseases, namely infectious diarrhea other than cholera, dysentery, typhoid, and paratyphoid. It has been noted in the literature that nosocomial norovirus infection usually involves frail older patients who may have prolonged symptoms [39]. As we age, the immune system and gut microbiota undergo significant changes in composition and function. These changes are associated with increased susceptibility to infectious diseases and decreased vaccination responses [40]. The dynamic microbial community of the human gastrointestinal tract plays a crucial role in health processes and supports the development and function of the intestinal immune barrier [41]. Furthermore, previous findings suggest that aging-associated microbiota can promote intestinal permeability and inflammation and eventually increase the levels of frailty-related proinflammatory cytokines [42,43]. All of this evidence suggests an association between frailty and infectious diseases, and our study further confirms this relationship.

In our study, we did not find an association between frailty and blood-borne, sexually transmitted, and contact-transmitted diseases. The prevalence of frailty in patients infected by HIV is high [44]. Research by Dias et al [45] showed that sex hormone-binding globulin is higher in patients with HIV and HCV, and that sex hormone-binding globulin is associated with aging-related conditions, such as osteoporosis and frailty in the general population. However, this effect was not observed in this study, and we hypothesize that this may have been due to the fact that we collected a larger number of cases of viral hepatitis and syphilis, and the existence of a latent period for these types of diseases and the difficulty in determining the true time of onset of the disease led to this result.

### Limitations

Our study has the following limitations. First, we only collected 235 cases with 12 infectious diseases, and these did not cover all types of infectious diseases. Second, our assessment of physical activity levels was obtained by self-reports, which may have introduced recall bias. Third, due to the impact of the changes in COVID-19 prevention and control measures, the cases we collected in the Chinese Disease Control and Prevention Information System-Infectious Disease Monitoring and Public Health Emergency Monitoring System did not include all infected persons, but rather those who were identified in hospitals by nucleic acid or antigen testing and reported to the Chinese Disease Control and Prevention Information System-Infectious Disease Monitoring and Public Health Emergency Monitoring System. Therefore, future studies using larger cohort data are necessary to further explore and replicate the results of this study.

### Conclusions

In summary, our population-based study suggests that frailty is associated with a higher risk of infectious disease in community-dwelling older adults aged 65 years and older. In the current era of global aging, the health of the older population is receiving more attention. Infectious diseases, in turn, are recognized as a major cause of global health loss, as well as the susceptibility of older adults to them. Therefore, in order to reduce the risk and burden of infectious diseases, the assessment of frailty in older adults should be added to future efforts.

## Supplementary material

10.2196/59762 Multimedia Appendix 1 Cox proportional risk regression modeling of the association between frailty subgroups and infectious disease risk among the elderly population in Dongguan (2018-2023)

## Abbreviations

HCV: hepatitis C virus
HR: hazard ratio
IRR: incidence rate ratio
