# Correction: Assessing the Impact of Frailty on Infection Risk in Older Adults: Prospective Observational Cohort Study

**DOI:** 10.2196/78866

**Published:** 2025-06-19

**Authors:** Ya Yang, Kechun Che, Jiayan Deng, Xinming Tang, Wenyuan Jing, Xiuping He, Jiacheng Yang, Wenya Zhang, Mingjuan Yin, Congcong Pan, Xiaoling Huang, Zewu Zhang, Jindong Ni

**Affiliations:** 1 School of Public Health, Shunde Women and Children’s Hospital, Precision Key Laboratory of Public Health, Guangdong Medical University, No.1 Xincheng Road, Dongguan, 523808, China, 86 15817668208; 2 Office of Public Health, Songshan Lake Community Health Service Centre, DongGuan, China; 3 Institute for Infectious Disease Prevention and Control, DongGuan Centre for Disease Control and Prevention, DongGuan, China

In “Assessing the Impact of Frailty on Infection Risk in Older Adults: Prospective Observational Cohort Study” (JMIR Public Health Surveill. 2024;10:e59762) the authors noted one error.

Affiliations 1 and 2 for authors Ya Yang, Kechun Che, Jiayan Deng, Xinming Tang, Wenyuan Jing, Xiuping He, Jiacheng Yang, Wenya Zhang, Mingjuan Yin, Congcong Pan, and Jindong Ni have been combined, from:

*School of Public Health, Shunde Women and Children’s Hospital, Foshan, China*

And:

*Precision Key Laboratory of Public Health, Guangdong Medical University, DongGuan, China*

To read as:

*School of Public Health, Shunde Women and Children’s Hospital, Precision Key Laboratory of Public Health, Guangdong Medical University, Dongguan, China*

The correction will appear in the online version of the paper on the JMIR Publications website, together with the publication of this correction notice. Because this was made after submission to PubMed, PubMed Central, and other full-text repositories, the corrected article has also been resubmitted to those repositories.

