# Supplementary material for: Assessing the Impact of Frailty on Infection Risk in Older Adults: Prospective Observational Cohort Study
Source: JMIR Public Health Surveill. 2024 Oct 16;10:e59762. doi: 10.2196/59762 (PMC11498063; doi:10.2196/59762)
Supplement: Multimedia Appendix 1 [file publichealth-v10-e59762-s001.doc]

| **Groups** | **Total person-years,n** | **Cases,n** | **Incidence density(/ 100000 person-years),n** | **IRR(95%CI)** |
| --- | --- | --- | --- | --- |
| **Frailty** | 3916.16 | 43 | 1098.01 | 2.09(1.44-2.99) |
| **Pre-frailty** | 8663.68 | 71 | 819.51 | 1.56(1.15-2.11) |
| **Health** | 23078.66 | 121 | 524.29 | 1(reference) |
| **Total** | 35658.50 | 235 | 659.03 | N/A^b^ |
| ^a^IRR:incidence rate ratio.  ^b^N/A:not applicable. | | | | |

Table1 Person-years, cases, incidence density, and IRR^a^ of older adults with frailty divided into three groups in a population-based cohort study of older adults in Dongguan, 2018‐2023.

Table S2 The outcomes of Cox proportional hazards regression models assessing the association between the frailty divided into three groups and the risk of infectious disease adjusted for age, gender, community participation status, exercise status(Dongguan; 2018‐2023; N=11,930)

| **Factor** | ***P value*** | **HR(95%CI)** |
| --- | --- | --- |
| **Group** |  |  |
| Health | Reference |  |
| Pre-frailty | .02 | 1.45(1.07-1.96) |
| Frailty | .01 | 1.65(1.12-2.44) |
| **Age(year)** |  |  |
| 65- | Reference |  |
| 75- | <.001 | 1.97(1.48-2.62) |
| 85- | .047 | 1.71(1.01-2.90) |
| **Gender** |  |  |
| Women | Reference |  |
| Men | <.001 | 2.03(1.49-2.76) |
| **Participation in community activities** |  |  |
| Never | Reference |  |
| Frequent | .03 | 0.67(0.47-0.97) |
| Ocassional | .68 | 1.10(0.72-1.68) |
| **Exercise** |  |  |
| High intensity | Reference |  |
| Low intensity | .27 | 1.28(0.83-1.97) |
| Medium intensity | .003 | 1.70(1.20-2.417) |

Table S3 The outcomes of Cox proportional hazards regression models assessing the association between the frailty and the risk of infectious disease(excluding hepatitis B and C and tuberculosis) adjusted for age, gender, exercise status (Dongguan; 2018‐2023; N=11,891)

| **Factor** | *P value* | **HR(95%CI )** |
| --- | --- | --- |
| **Group** |  |  |
| Health | Reference |  |
| Frailty | <.001 | 1.83(1.36-2.46) |
| **Age(year)** |  |  |
| 65- | Reference |  |
| 75- | <.001 | 1.98(1.46-2.69) |
| 85- | .01 | 1.98(1.18-3.30) |
| **Gender** |  |  |
| Women | Reference |  |
| Men | <.001 | 2.05(1.54-2.72) |
| **Exercise** |  |  |
| High intensity | Reference |  |
| Low intensity | .13 | 1.45(0.90-2.32) |
| Medium intensity | <.001 | 1.95(1.32-2.88) |

Table S4 The outcomes of Cox proportional hazards regression models assessing the association between the frailty divided into three groups and the risk of infectious disease(excluding hepatitis B and C and tuberculosis) adjusted for age, gender, community participation status, exercise status(Dongguan; 2018‐2023; N=11,891)

| **Factor** | ***P value*** | **HR(95%CI )** |
| --- | --- | --- |
| **Group** |  |  |
| Health | Reference |  |
| Pre-frailty | .001 | 1.78(1.29-2.46) |
| Frailty | .003 | 1.89(1.23-2.89) |
| **Age(year)** |  |  |
| 65- | Reference |  |
| 75- | <.001 | 2.02(1.47-2.77) |
| 85- | .01 | 2.05(1.19-3.53) |
| **Gender** |  |  |
| Women | Reference |  |
| Men | <.001 | 2.16(1.54-3.03) |
| **Tobacco use** |  |  |
| Never | Reference |  |
| Ever | .22 | 0.75(0.48-1.19) |
| Yes | .049 | 0.69(0.48-1.00) |
| **Exercise** |  |  |
| High intensity | Reference |  |
| Low intensity | .15 | 1.42(0.88-2.89) |
| Medium intensity | .003 | 1.83(1.23-2.71) |

Table S5 The outcomes of Cox proportional hazards regression models assessing the association between the frailty and the risk of infectious disease adjusted for age, sex, community participation status, exercise status, diabetes , stroke (Dongguan; 2018‐2023; N=11,930).

| **Factor** | ***P value*** | **HR(95%CI )** |
| --- | --- | --- |
| **Group** |  |  |
| Health | Reference |  |
| Frailty | .01 | 1.42(1.08-1.87) |
| **Age(year)** |  |  |
| 65- | Ref |  |
| 75- | <.001 | 1.97(1.50-2.60) |
| 85- | .02 | 1.83(1.10-3.02) |
| **Gender** |  |  |
| Women | Reference |  |
| Men | <.001 | 1.90(1.47-2.47) |
| **Participation in community activities** |  |  |
| Never | Reference |  |
| Frequent | .02 | 0.65(0.45-0.93) |
| Ocassional | .56 | 1.13(0.74-1.73) |
| **Exercise** |  |  |
| High intensity | Reference |  |
| Low intensity | .29 | 1.26(0.82-1.93) |
| Medium intensity | .002 | 1.73(1.22-2.44) |
| **Diabetes** |  |  |
| No | Reference |  |
| Yes | .01 | 1.61(1.16-2.24) |
| **Stroke** |  |  |
| No | Reference |  |
| Yes | .02 | 2.52(1.18-5.41) |
